# Frequency and gender differences in the use of professional home care in late life. Findings from three German old-age cohorts

**DOI:** 10.3389/fmed.2022.924818

**Published:** 2022-09-02

**Authors:** Elżbieta W. Buczak-Stec, André Hajek, Alexander Pabst, Christian Brettschneider, Hendrik van den Bussche, Birgitt Wiese, Siegfried Weyerer, Jochen Werle, Andreas Hoell, Michael Pentzek, Angela Fuchs, Melanie Luppa, Margit Löbner, Janine Stein, Franziska Förster, Dagmar Weeg, Edelgard Mösch, Kathrin Heser, Martin Scherer, Wolfgang Maier, Matthias C. Angermeyer, Michael Wagner, Steffi G. Riedel-Heller, Hans-Helmut König

**Affiliations:** ^1^Department of Health Economics and Health Services Research, Hamburg Center for Health Economics, University Medical Center Hamburg-Eppendorf, Hamburg, Germany; ^2^Institute of Social Medicine, Occupational Health and Public Health, University of Leipzig, Leipzig, Germany; ^3^Department of General Practice and Primary Medical Care, Center for Psychosocial Medicine, University Medical Center Hamburg-Eppendorf, Hamburg, Germany; ^4^Institute of General Practice, Hannover Medical School, Hannover, Germany; ^5^Central Institute of Mental Health, Medical Faculty Mannheim/Heidelberg University, Mannheim, Germany; ^6^Institute of General Practice (ifam), Centre for Health and Society (chs), Medical Faculty, Heinrich Heine University Düsseldorf, Düsseldorf, Germany; ^7^Department of Psychiatry, Technical University of Munich, Munich, Germany; ^8^Department of Neurodegenerative Diseases and Geriatric Psychiatry, University Hospital Bonn, Bonn, Germany; ^9^German Center for Neurodegenerative Diseases (DZNE), Bonn, Germany; ^10^Center for Public Mental Health, Gösing am Wagram, Austria

**Keywords:** gender differences, professional home care, home-based formal help, home care, outpatient nursing care, meals on wheels, healthcare utilization

## Abstract

**Aim:**

The aim of this study was to investigate the frequency of and the gender differences in the use of professional home care in Germany.

**Methods:**

We used harmonized data from three large cohort studies from Germany (“Healthy Aging: Gender-specific trajectories into the latest life”; AgeDifferent.de Platform). Data were available for 5,393 older individuals (75 years and older). Mean age was 80.2 years (SD: 4.1 years), 66.6% were female. Professional homecare outcome variables were use of outpatient nursing care, paid household assistance, and meals on wheels' services. Logistic regression models were used, adjusting for important sociodemographic variables.

**Results:**

Altogether 5.2% of older individuals used outpatient nursing care (6.2% women and 3.2% men; *p* < 0.001), 24.2% used paid household assistance (26.1% women and 20.5% men; *p* < 0.001) and 4.4% used meals on wheels' services (4.5% women and 4.0% men; *p* = 0.49). Regression analysis revealed that women had higher odds of using paid household assistance than men (OR = 1.48, 95% CI: [1.24–1.76]; *p* < 0.001), whereas they had lower odds of using meals on wheels' services (OR = 0.64, 95% CI: [0.42–0.97]; *p* < 0.05). No statistically significant differences in using outpatient nursing care between women and men were found (OR = 1.26, 95% CI: [0.87–1.81]; *p* = 0.225). Further, the use of home care was mainly associated with health-related variables (e.g., stroke, Parkinson's disease) and walking impairments.

**Conclusions:**

Our study showed that gender differences exist in using paid household assistance and in culinary dependency. For example, meals on wheels' services are of great importance (e.g., for individuals living alone or for individuals with low social support). Gender differences were not identified regarding outpatient nursing care. Use of professional home care services may contribute to maintaining autonomy and independence in old age.

## Introduction

The number and proportion of older individuals in many developed societies will increase considerably in the coming decades. As old age is associated with an increasing need for care (1–4), the number of individuals in need of care will presumably increase. These individuals usually prefer to be cared for at home, e.g., to maintain social contacts for as long as possible (5). An important resource with presumably increasing relevance is thus professional home care.

Professional home care encompasses multidisciplinary care which is provided by e.g., health professionals to individuals in their own homes (3, 6). It may embrace outpatient nursing care, paid household assistance and also other services such as meals on wheels. The main advantage of providing home care services is that they can assist in maintaining independence and may contribute to reducing the risk of hospital admissions or nursing home placement (6, 7). Moreover, it has been shown that quality of life of the individuals in need of care can be increased through being cared at home (6). Other professional home care services can also be of great benefit for older individuals with e.g., culinary dependency–a support need, when someone is not able to prepare all meals by themselves (8, 9). Culinary dependency is commonly associated with malnutrition (8) which in turn may have adverse health consequences (10, 11). Using meals on wheels' services may thus be beneficial for older individuals at risk of poor nutrition.

The use of the professional home care and other community support services is influenced by variety of factors (12–16). The majority of these factors are health related, including morbidity, walking disabilities, visual impairments, and stroke history (12, 15, 17). In addition, the use of professional home care is known to increase with age (12, 16). Furthermore, previous studies showed that social factors, especially living alone or having children, are important factors that influence use of the services (12, 16, 18–20). Importantly, those factors may influence women to a greater extent than men, owing to greater life expectancy, higher probability of being widowed, different health status, and also different needs for community services (14, 18). Considering those facts and previous research, we hypothesized that gender differences in use of professional home care will be evident. We hypothesized that women will use the services more often.

Thus far, there is restricted knowledge regarding the frequency and potential gender differences in professional home care in later life. For example, a previous study examined the factors related to such services among older adults in Germany (17). It showed that using such services is associated with health-related factors. However, this previous study had a different focus [more generally on informal and formal caregiving time and was restricted to one cohort study (17)].

In sum, there is limited knowledge regarding the frequency and possible gender differences with regard to professional home care. The knowledge about the frequency and determinants of use of professional home-based care and potential gender differences is essential since those services are of great advantage for older individuals. Using those services may contribute to extended independent living in the community. Therefore, based on harmonized data from three large cohorts of older individuals in Germany, the aim of this study was (i) to investigate the frequency and determinants of use of professional home care and (ii) to explore potential gender differences in professional home care (in terms of outpatient nursing care, paid household assistance, and meals on wheels' services).

## Methods

### Study design and participants

In this study, we analyzed harmonized baseline data from the platform “Healthy Aging: Gender-specific trajectories into the latest life” (AgeDifferent.de platform). The AgeDifferent.de platform consists of harmonized prospective cohort studies from three large representative German primary care based old-age cohorts, namely “German Study on Aging, Cognition, and Dementia in Primary Care Patients” (AgeCoDe/AgeQualiDe), “Late-life depression in primary care: needs, health care utilization and costs” (AgeMooDe) and, “Leipzig Longitudinal Study of the Aged” (LEILA75+). AgeCoDe/AgeQualiDe is a large multicenter prospective cohort study of primary care patients aged 75 years and more. Baseline data and data of nine follow-ups assessments (FU) were collected between 2003 and 2017. AgeMooDe is a longitudinal, multicentre, prospective cohort study that collected data from four centers in Germany (Bonn, Hamburg, Leipzig, and Mannheim). In the AgeMooDe study, participants (aged 75 years or older) were recruited through practices of general practitioners. Baseline data were collected from May 2012 to December 2013. For both studies, trained staff conducted a standardized clinical interview in participants' homes. LEILA75+ is a study on the epidemiology of dementia and mild cognitive impairment (MCI) among community dwelling individuals aged 75 years and older. This random sample of participants was recruited from the local registry office in Leipzig. The study consisted of baseline data and five follow-ups scheduled every 18 months. More detailed information about each cohort is provides by the following publications [LEILA 75+ (21), AgeCoDe/AgeQualiDe (22, 23), AgeMooDe (24)].

Within the AgeDifferent.de platform, in sum 6,470 participants aged 75 years and older with over 26,000 person-years can be examined. After excluding individuals with missing data (25), altogether n=5,745 individuals from the baselines of three cohorts were included in the present study (LEILA 75+ *n* = 1,265, AgeCoDe *n* = 3,287; and AgeMooDe *n* = 1,193).

The AgeDifferent.de platform represents a unique resource for investigating aging-related issues among older adults in Germany. The pooling of data from three large cohorts allows for more accurate analyses and the possibility to investigate certain subgroups (e.g., home care utilization of older individuals stratified by education or marital status). Further information about harmonized data included in the AgeDifferent.de platform are provided by Förster et al. (25).

A written informed consent was obtained from all participants in the three original cohort studies prior to study participation. Ethics committees at all participating centers approved each of the original studies.

### Dependent variables

Our primary outcome of interest was utilization of professional home care among older individuals in terms of using outpatient nursing care, paid household assistance, and culinary dependency. At baseline of each of studies, participants were asked “Have you used outpatient nursing care in the last 3 months?” (yes; no) and “Have you used paid household assistance in the last 3 months?” (yes; no). Culinary dependence was defined in term of the use of the Meals on Wheels service (yes; no).

### Covariates

In our analysis, we adjusted for important sociodemographic variables that are linked to the use of home care and long-term care (26, 27). We included sex, age, marital status (single; married; divorced; widowed), living situation (living alone, living with a spouse, a relative or other person; living in nursing home), children (yes; no) and educational level. Education was categorized according to the highest academic qualification (no vocational training; technical training; university; other degree). In our analysis, we also included risk factors associated with home care (12–16)–history of stroke, TIAs or related syndromes, cardiac problems (including infraction, CHD, arrhythmias), diabetes and Parkinson's disease. The presence of chronic diseases was assessed by primary care physicians. Each variable was dichotomized (yes; no). In addition, walking impairments, vision impairments, and hearing impairments were included. The variables were dichotomized in (i) no impairments; and (ii) difficulties, considerable restrictions or impaired). Since we used harmonized data, we further controlled for the study in which the participant took part. Controlling for the individual studies used in the harmonized data is also of importance, as the baseline data in each study refers to different time point of time.

### Statistical analysis

We described the characteristics of participants at baseline (harmonized data from three cohorts). The characteristics were stratified by sex and the differences were compared using non-parametric or χ^2^ test, as appropriate. In order to assess the gender differences in utilization of professional home care we used multivariate logistic regression models. Results are presented as the point estimates - Odds Ratios and 95% confidence intervals (OR, 95% CI). In a sensitivity analysis, we estimated models with Firth logistic regression (28, 29) in order to adjust for possible sparse data bias. We also conducted a subgroup analysis. Statistical significance was defined as *p* value of 0.05 or smaller. Analyses were conducted using Stata 16.1 (StataCorp., College Station, Texas, USA).

## Results

### Study design and participants

Our analytical sample consisted of 5,305 older individuals that provided information about use of outpatient nursing care, use of paid household assistance and other sociodemographic control variables (Table 1). Data about using meals on wheels' services were available for *n* = 4,250 older individuals (for LEILA 75+ and AgeCoDe, but not for AgeMooDe).

**Table 1:** Descriptive statistics of the analytical sample at baseline for the pooled data AgeDifferent.de platform.

**Variables**	**Women**	**Men**	**Total sample**	
**Mean (SD); range/N (%)**	**(*n* = 3,530)**	**(*n* = 1,775)**	**(*n* = 5,305)**	***p*-values**
Age	80.39 (4.12); 75–99	79.93 (4.05); 75–99	80.24 (4.10); 75–99	<0.001
Sex				
- Female	3,530 (100.0%)	-	3,530 (66.5%)	
- Male	-	1,775 (100.0%)	1,775 (33.5%)	
Educational level				<0.001
- No vocational training	1,142 (32.4%)	118 (6.6%)	1,260 (23.8%)	
- Technical training	2,077 (58.8%)	1,207 (68.0%)	3,284 (61.9%)	
- University degree	233 (6.6%)	419 (23.6%)	652 (12.3%)	
- Other degree	78 (2.2%)	31 (1.7%)	109 (2.1%)	
Marital status				<0.001
- Single	278 (7.9%)	42 (2.4%)	320 (6.0%)	
- Married	877 (24.8%)	1,294 (72.9%)	2,171 (40.9%)	
- Divorced	280 (7.9%)	59 (3.3%)	339 (6.4%)	
- Widowed	2,095 (59.3%)	380 (21.4%)	2,475 (46.7%)	
Children				<0.001
- No	744 (21.1%)	266 (15.0%)	1,010 (19.0%)	
- Yes	2,786 (78.9%)	1,509 (85.0%)	4,295 (81.0%)	
Living situation				<0.001
- Living alone	2,330 (66.0%)	392 (22.1%)	2,722 (51.3%)	
- Living with a spouse, relative or other person	1,096 (31.0%)	1,355 (76.3%)	2,451 (46.2%)	
- Living in nursing home	104 (2.9%)	28 (1.6%)	132 (2.5%)	
Cardiac problems (infraction, CHD)	1,299 (36.8%)	927 (52.2%)	2,226 (42.0%)	<0.001
History of stroke	303 (8.6%)	252 (14.2%)	555 (10.5%)	<0.001
Diabetes	841 (23.8%)	468 (26.4%)	1,309 (24.7%)	0.04
Parkinson's Disease	49 (1.4%)	37 (2.1%)	86 (1.6%)	0.06
Vision impairment	767 (21.7%)	286 (16.1%)	1,053 (19.8%)	<0.001
Walking impairment	1,491 (42.2%)	608 (34.3%)	2,099 (39.6%)	<0.001
Hearing impairment	1,059 (30.0%)	717 (40.4%)	1,776 (33.5%)	<0.001
**Outcome variables**
Outpatient nursing care				<0.001
- No	3,309 (93.8%)	1,717 (96.8%)	5,026 (94.8%)	
- Yes	220 (6.2%)	57 (3.2%)	277 (5.2%)	
Paid household assistance				<0.001
- No	2,607 (73.9%)	1,412 (79.5%)	4,019 (75.8%)	
- Yes	923 (26.1%)	363 (20.5%)	1,286 (24.2%)	
Culinary dependency^a^				0.49
- No	2,735 (95.5%)	1,328 (96.0%)	4,063 (95.6%)	
- Yes	129 (4.5%)	56 (4.0%)	185 (4.4%)	
Cohort studies:
LEILA 75+	735 (20.8%)	258 (14.5%)	993 (18.7%)	
AgeCoDe/AgeQualiDe	2,130 (60.3%)	1,128 (63.5%)	3,258 (61.4%)	
AgeMooDe	665 (18.8%)	389 (21.9%)	1,054 (19.9%)	

Data stratified by gender.

SD, standard deviation; p-values based on chi-square test and non-parametric tests; ^a^data only available for two cohorts LEILA 75+ and AgeCoDe.

Mean age of study participants was 80.2 years (SD 4.1) ranging from 75 to 99 years, 66.5% were female, 40.9% were married, 46.7% were widowed, and 51.3% lived alone. Altogether 5.2% older individuals used outpatient nursing care, paid household assistance was used by 24.2% of individuals and 4.4% were using the meals on wheels' services.

In our sample, women were older than men [80.4 years (SD: 4.1) vs. 79.9 (SD: 4.1); *p* < 0.001] and they differed with regard to educational level (women had lower education level in comparison to men; *p* < 0.001), marital status (*p* < 0.001), and living situation (*p* < 0.001). For example, women were more frequently widowed than men (59.2 vs. 21.3%) and were more likely to live alone (66.0 vs. 22.1%). Furthermore, the frequency of using various professional home care services in late life varied with regard to sex. Women used the outpatient nursing care (6.4 vs. 3.2%; *p* < 0.001) and paid household assistance more frequently than men (26.1 vs. 20.5%; *p* < 0.001). There were no significant differences between women and men in using the meals on wheels' services (4.5 vs. 4.0%; *p* = 0.49).

### Results of regression analyses

After controlling for important sociodemographic variables, the result of logistic regressions revealed that there were significant gender differences in use of paid household assistance and in the culinary dependency among older individuals (Table 2). Namely, women had higher odds of using paid household assistance than men (OR = 1.50, 95% CI: [1.26–1.77]; *p* < 0.001). In contrast, women had lower odds of using the meals on wheels' services than men (OR = 0.65, 95% CI: [0.44–0.97]; *p* < 0.05). We did not find statistically significant differences in using outpatient nursing home between women and men (OR = 1.27, 95% CI: [0.90–1.78]; *p* = 0.175).

**Table 2:** Results of the multivariate logistic regressions.

	**Outpatient nursing care**		**Paid household assistance**		**Culinary dependence (meals on wheels)**	
**Variables**	**OR**	**95% CI**	**OR**	**95% CI**	**OR**	**95% CI**
Female (ref. male)	1.26	(0.87–1.81)	1.48***	(1.24–1.76)	0.64*	(0.42–0.97)
Age	1.05**	(1.02–1.08)	1.08***	(1.06–1.10)	1.09***	(1.05–1.13)
Educational level (ref. no vocational training)						
- Technical training	0.95	(0.70–1.27)	1.37***	(1.16–1.62)	1.05	(0.72–1.55)
- University degree	0.75	(0.43–1.31)	3.40***	(2.66–4.34)	1.55	(0.86–2.79)
- Other degree	1.13	(0.48–2.67)	1.94**	(1.25–3.03)	3.97**	(1.64–9.62)
Marital status (ref. single)						
- Married	0.84	(0.39–1.77)	1.28	(0.89–1.84)	0.82	(0.35–1.93)
- Divorced	1.68	(0.80–3.53)	1.44+	(0.98–2.11)	1.70	(0.75–3.86)
- Widowed	1.63	(0.87–3.04)	1.51**	(1.12–2.05)	1.49	(0.75–2.95)
Children (ref. no)	0.76	(0.54–1.08)	0.61***	(0.51–0.72)	0.60**	(0.41–0.88)
Living situation (ref. living with a spouse, relative or other person)						
- Living alone	1.19	(0.78–1.83)	1.19	(0.94–1.51)	1.48	(0.87–2.49)
- Living in nursing home	3.89***	(2.22–6.80)	1.40	(0.91–2.16)	3.30**	(1.48–7.34)
Cardiac problems (infraction, CHD) (ref. no)	1.30+	(0.97–1.74)	1.23**	(1.06–1.42)	1.95***	(1.32–2.89)
History of stroke (ref. no)	1.72**	(1.22–2.42)	1.06	(0.86–1.32)	2.07**	(1.33–3.21)
Diabetes (ref. no)	1.72***	(1.31–2.25)	1.06	(0.90–1.24)	0.90	(0.62–1.31)
Parkinson's Disease (ref. no)	3.32***	(1.76–6.24)	1.46	(0.90–2.37)	1.03	(0.31–3.43)
Vision impairment (ref. no)	1.15	(0.86–1.53)	1.31**	(1.11–1.55)	1.44*	(1.02–2.06)
Walking impairment (ref. no)	7.01***	(4.91–10.00)	2.07***	(1.80–2.38)	2.47***	(1.76–3.47)
Hearing impairment (ref. no)	1.04	(0.79–1.36)	1.09	(0.95–1.26)	1.00	(0.72–1.39)
Study centre	Yes		Yes		Yes	
Constant	0.00***	(0.00–0.00)	0.00***	(0.00–0.00)	0.00***	(0.00–0.00)
Observations	5,305		5,305		4,250^a^	

Gender differences in utilization of professional home care.

OR 95% CI, Odds Ratios with 95% confidence intervals; ^a^data only available for two cohorts LEILA 75+ and AgeCoDe.

***p < 0.001, **p < 0.01, *p < 0.05, +p < 0.1.

In addition, the analyses showed that the use of *all* professional home care services increased with age (OR = 1.05, 95% CI: [1.02–1.08], *p* < 0.01 for outpatient nursing care; OR = 1.08, 95% CI: [1.06–1.10], *p* < 0.001 for paid household assistance and OR = 1.09, 95% CI: [1.05–1.13], *p* < 0.001 for culinary dependency). Moreover, compared with individuals without impairments, individuals with walking impairments had higher odds of using home care, namely using outpatient nursing care (OR = 7.01, 95% CI: [4.9–10.0]; *p* < 0.001), paid household assistance (OR = 2.07, 95% CI: [1.80–2.38], *p* < 0.001) and meals on wheels (OR = 2.47, 95% CI [1.76–3.47]).

Individuals with children were less likely to use paid household assistance (OR = 0.61, 95% CI: [0.51–0.72] *p* < 0.001) and meals on wheels (OR = 0.60, 95% CI: [0.41–0.88], *p*< *0.01*). Higher odds of using outpatient nursing care were among individuals with history of stroke (OR = 1.72, 95% CI: [1.22–2.42], *p* < −0.01), diabetes (OR = 1.72 95% CI: [1.31–2.25], *p* < 0.001), or Parkinson's disease (OR = 3.32, 95% CI: [1.76–6.24], *p* < 0.001).

In sensitivity analysis, we estimated the models using Firth logistic regressions. In terms of sex differences and main determinants of use of professional home care, the results remained virtually the same regarding effect size and significance (Supplementary Table 1). In another sensitivity analysis, we included only the subgroup of individuals living alone. With the exception of gender differences in the use of paid household assistance (gender differences were not statistically significant OR = 1.07 95% CI: [0.82–1.39]), other results remained the same (Supplementary Table 2).

## Discussion

The aim of this study was to investigate the frequency of use and potential gender differences in professional home care (in terms of outpatient nursing care, paid household assistance, and using the meals on wheels' services) in Germany. Paid household assistance was most frequently used, followed at a greater distance by outpatient nursing care and meals on wheels' services–altogether with little gender differences. Regressions showed that women had higher odds of using paid household assistance than men, whereas they had lower odds of using the meals on wheels' services. No statistically significant differences in using outpatient nursing home were identified between women and men. Our current study adds to our very limited current knowledge by identifying some gender differences between professional home care services among older adults in Germany.

In our study, men had a higher likelihood of using meals on wheels' services than women. This was also evident in the subgroup of individuals living alone. This is in line with a previous study showing that men who lived alone sought after support particularly in preparing food (14). Women may underutilize those services due to gender-specific reasons. It may be possible, that women have difficulties in accepting meals that are not prepared by themselves. The differences in utilization may be also explained through a well-known classic allocation of roles in the household in these birth cohorts. While women were commonly responsible for the household, men were often responsible for the livelihoods in Germany (in such birth cohorts). Thus, these gender differences may be explained by differences in cooking skills between both sexes. It may be interesting to examine such potential gender differences in these outcomes in upcoming decades (and consequently more recent birth cohorts). Moreover, meals on wheels' services also have a social dimension (30, 31). For example, a qualitative study (32) showed that such a service can provide meals recipients with social contact–and we assume that particularly older men [who are often less likely to have developed friendships (32)] may more frequently yearn to see someone–at least shortly as in the case of meals on wheels' services. In contrast, women had a higher likelihood of using a paid household assistance in our study. This may be explained by differences in help-seeking behavior (33–35). It might be also possible that men underutilized these services, as they are not accustomed to accepting help. Apart from inevitable basic care, men may thus try to avoid using paid household assistances, while women may be more open-minded to using such services. It is also possible the paid household assistance services do not have a very high priority for men or that they do not have a great need for such as services. Moreover, in our study we could not control for the size or type of home in which the individuals lived. It is possible, that women are more likely to live in larger homes, which could lead to a greater need for support. Additionally, when only the subgroup of individuals living alone was analyzed, the gender differences were weaker and did not achieve statistical significance.

Interestingly, gender differences were not identified regarding outpatient nursing care in our study. We assume that both sexes are aware of the great need of such basic services to avoid nursing or old age home admission for as long as possible–which meets the preferences of most older individuals in Germany (36).

Moreover, apart from increasing age, we showed that individuals with walking impairments were more likely to use all types of home care services, namely outpatient nursing care, paid household assistance and they were also more likely to be culinary dependent. The present findings are consistent with other research which found that worse functioning or disability are strongly associated with increased care needs, which may further contribute to using home and subsequently, to eventual nursing home placement (12, 19, 20, 26). We also showed that individuals with history of stroke, diabetes and Parkinson's' disease were important factors that conditioned high use of outpatient nursing care. These results align with the findings of other studies, in which, among others, history of stroke, multimorbidity and low health status were associated with higher utilization of home care services (12, 15, 18, 19). The gender differences in the use of professional home care and the low utilization of some of these services (e.g., outpatient nursing care as well as meals on wheels services) may also reflect a possible inadequate provision of home care in older age. However, this hypothesis should be further explored in further research.

Some strengths and limitations of our current study are worth mentioning. A key strength is that we used harmonized data from three large and well-known cohort studies (also including oldest olds). Furthermore, main professional home care services were included in our study. It should be noted that some selection bias has been identified in the respective studies [e.g., (37)]. Furthermore, our results reflect the use of professional home care at baseline in all studies. Changes in professional home care utilization and the current use should be further investigated in longitudinal studies. In our analysis we could not control for the number of individuals who lived the household and the size of home. However, we adjusted for the housing situation (living alone, with spouse, with relatives or in nursing home). Moreover, culinary dependency was only available in two out of the three cohorts.

In conclusion, our study showed that some gender differences exist in using paid household assistance and in culinary dependency (but not in using outpatient nursing care). For example, meals on wheels' services are of great importance (e.g., for individuals living alone or for individuals with low social support). Knowledge about such gender differences can potentially assist in avoiding malnutrition among individuals in late life. More generally, it may assist in avoiding unmet needs. Use of professional home care services may contribute to maintaining autonomy and independence in old age. However, these outcomes may also be adversely affected by potential gender inequality. Future research from other countries is required to better understand the frequency and potential gender differences in professional home care.

## Data availability statement

The datasets presented in this article are not readily available because ethical restrictions involving patient data. Underlying data are available upon request from the Working Group Medical Statistics and IT-Infrastructure. Requests to access the datasets should be directed to BW, wiese.birgitt@mh-hannover.de.

## Ethics statement

The studies involving human participants were reviewed and approved by the Ethics Committee of all participating centres. The patients/participants provided their written informed consent to participate in this study.

## Author contributions

EB-S and AHa made substantial contributions to the conception and design of the study, the analysis and interpretation of data, and drafted the manuscript. H-HK and SR-H made substantial contributions to the interpretation of data, drafting of the manuscript, and were responsible for supervision. AP, CB, HB, BW, SW, JW, AHo, MP, AF, MLu, MLö, JS, FF, DW, EM, KH, MS, WM, MA, and MW made substantial contributions. All authors contributed to the article and approved the submitted version.

## Funding

This publication is part of the study “Healthy Aging: Gender-specific trajectories into latest life” (AgeDifferent.de) and was funded by the German Federal Ministry of Education and Research (Funding program “Gesund—-ein Leben lang”) (grants 01GL1714A, 01GL1714B, 01GL1714C, and 01GL1714D).

## Conflict of interest

The authors declare that the research was conducted in the absence of any commercial or financial relationships that could be construed as a potential conflict of interest.

## Publisher's note

All claims expressed in this article are solely those of the authors and do not necessarily represent those of their affiliated organizations, or those of the publisher, the editors and the reviewers. Any product that may be evaluated in this article, or claim that may be made by its manufacturer, is not guaranteed or endorsed by the publisher.
