# Supplementary material for: Frequency and gender differences in the use of professional home care in late life. Findings from three German old-age cohorts
Source: Front Med (Lausanne). 2022 Sep 2;9:924818. doi: 10.3389/fmed.2022.924818 (PMC9478461; doi:10.3389/fmed.2022.924818)
Supplement: Supplementary file 1 [file Table_1.pdf]

Supplementary data

Sensitivity analysis

Frequency and gender differences in the use of professional home care in late life. Findings from three German old-age cohorts

Elżbieta W. Buczak-Stec, André Hajek, Alexander Pabst, Christian Brettschneider, Hendrik van den Bussche, Birgitt Wiese, Siegfried Weyerer, Jochen Werle , Andreas Hoell, Michael Pentzek, Angela Fuchs, Melanie Luppa, Margit Löbner, Janine Stein, Franziska Förster, Dagmar Weeg, Edelgard Mösch, Kathrin Heser, Martin Scherer, Wolfgang Maier, Matthias C. Angermeyer, Michael Wagner, Steffi G. Riedel-Heller, Hans-Helmut König

Table S1 Sensitivity analysis. Results of the multivariate Firth logistic regressions. Gender differences in utilisation of professional home care.

	Outpatient nursing care		Paid household assistance		Culinary dependence (meals on wheels)	
VARIABLES	OR	95% CI	OR	95% CI	OR	95% CI
Female (ref. male)	1.25	(0.87 - 1.80)	1.47***	(1.23 - 1.76)	0.64*	(0.42 - 0.96)
Age	1.05**	(1.02 - 1.08)	1.08***	(1.06 - 1.10)	1.09***	(1.05 - 1.13)
Educational level (ref. no vocational training)						
- technical training	0.94	(0.70 - 1.27)	1.37***	(1.16 - 1.62)	1.05	(0.71 - 1.53)
- university degree	0.76	(0.44 - 1.32)	3.38***	(2.65 - 4.31)	1.55	(0.87 - 2.78)
- other degree	1.20	(0.52 - 2.75)	1.95**	(1.25 - 3.03)	4.11**	(1.74 - 9.70)
Marital status (ref. single)						
- married	0.81	(0.39 - 1.71)	1.27	(0.88 - 1.83)	0.79	(0.34 - 1.85)
- divorced	1.66	(0.80 - 3.44)	1.44+	(0.98 - 2.11)	1.68	(0.75 - 3.77)
- widowed	1.58	(0.85 - 2.92)	1.51**	(1.11 - 2.04)	1.44	(0.74 - 2.82)
Children (ref. no)	0.76	(0.54 - 1.07)	0.61***	(0.51 - 0.72)	0.60**	(0.41 - 0.87)
Living situation (ref. living with a spouse, relative or other person)						
- living alone	0.85	(0.55 - 1.29)	0.84	(0.66 - 1.06)	0.69	(0.41 - 1.16)
- living in nursing home	3.23***	(2.05 - 5.09)	1.18	(0.79 - 1.74)	2.26*	(1.15 - 4.42)
Cardiac problems (infraction, CHD) (ref. no)	1.29+	(0.97 - 1.74)	1.23**	(1.06 - 1.42)	1.94***	(1.31 - 2.86)
History of stroke (ref. no)	1.72**	(1.22 - 2.42)	1.06	(0.86 - 1.32)	2.07**	(1.34 - 3.20)
Diabetes (ref. no)	1.71***	(1.31 - 2.23)	1.06	(0.91 - 1.24)	0.91	(0.63 - 1.31)
Parkinson’s Disease (ref. no)	3.34***	(1.79 - 6.23)	1.47	(0.91 - 2.37)	1.18	(0.38 - 3.62)
Vision impairment (ref. no)	1.15	(0.87 - 1.53)	1.31**	(1.11 - 1.55)	1.44*	(1.02 - 2.04)
Waking impairment (ref. no)	6.88***	(4.83 - 9.79)	2.06***	(1.79 - 2.37)	2.45***	(1.74 - 3.43)
Hearing impairment (ref. no)	1.04	(0.79 - 1.36)	1.09	(0.95 - 1.26)	1.00	(0.72 - 1.39)
Study centre	yes		yes		yes	
Constant	0.00***	(0.00 - 0.00)	0.00***	(0.00 - 0.00)	0.00***	(0.00 - 0.00)
Observations	5,393		5,393		4,288[a]	

Notes: OR 95% CI - Odds Ratios with 95% confidence intervals; [a] data only available for two cohorts LEILA 75+ and AgeCoDe.
*** p<0.001, ** p<0.01, * p<0.05, + p<0.1

Table S2 Sensitivity analysis. Results of the multivariate Firth logistic regressions. Gender differences in utilisation of professional home care among individuals *living alone*.

	Outpatient nursing care		Paid household assistance		Culinary dependence (Meals on Wheels)	
VARIABLES	OR	95% CI	OR	95% CI	OR	95% CI
Female (ref. male)	1.49	(0.86 - 2.57)	1.07	(0.82 - 1.39)	0.41***	(0.25 - 0.68)
Age	1.08***	(1.04 - 1.12)	1.08***	(1.06 - 1.11)	1.10***	(1.06 - 1.16)
Educational level (ref. no vocational training)						
- technical training	0.91	(0.64 - 1.30)	1.38**	(1.12 - 1.70)	0.88	(0.56 - 1.37)
- university degree	0.70	(0.33 - 1.49)	3.96***	(2.82 - 5.57)	1.17	(0.54 - 2.54)
- other degree	1.27	(0.44 - 3.67)	1.58	(0.87 - 2.86)	4.17**	(1.57 - 11.13)
Marital status (ref. single)						
- married	0.43	(0.07 - 2.59)	2.01*	(1.10 - 3.67)	0.70	(0.15 - 3.26)
- divorced	1.32	(0.58 - 3.00)	1.60*	(1.04 - 2.46)	1.40	(0.58 - 3.34)
- widowed	1.32	(0.66 - 2.65)	1.71**	(1.21 - 2.43)	1.05	(0.50 - 2.20)
Children (ref. no)	0.78	(0.52 - 1.19)	0.61***	(0.49 - 0.76)	0.59*	(0.37 - 0.94)
Cardiac problems (infraction, CHD) (ref. no)	1.30	(0.90 - 1.88)	1.23*	(1.01 - 1.49)	2.17**	(1.33 - 3.56)
History of stroke (ref. no)	1.45	(0.90 - 2.34)	1.22	(0.91 - 1.65)	2.24**	(1.24 - 4.07)
Diabetes (ref. no)	1.84***	(1.32 - 2.57)	1.22+	(0.99 - 1.50)	0.94	(0.60 - 1.48)
Parkinson's Disease (ref. no)	2.72*	(1.07 - 6.93)	1.55	(0.76 - 3.15)	0.25	(0.01 - 4.14)
Vision impairment (ref. no)	1.11	(0.78 - 1.58)	1.24+	(1.00 - 1.54)	1.42	(0.92 - 2.18)
Waking impairment (ref. no)	6.52***	(4.20 - 10.11)	2.27***	(1.89 - 2.73)	3.38***	(2.20 - 5.21)
Hearing impairment (ref. no)	1.29	(0.92 - 1.81)	1.20+	(0.99 - 1.46)	1.01	(0.67 - 1.52)
Study centre	yes		yes		yes	
Constant	0.00***	(0.00 - 0.00)	0.00***	(0.00 - 0.00)	0.00***	(0.00 - 0.00)
Observations	2,721		2,722		2,252 [a]	

Notes: OR 95% CI - Odds Ratios with 95% confidence intervals; [a] data only available for two cohorts LEILA 75+ and AgeCoDe.
*** p<0.001, ** p<0.01, * p<0.05, + p<0.1
